# Testing the thermal physiology, habitat and competition hypotheses for elevational range limits in four tropical songbirds

**DOI:** 10.1098/rspb.2025.1953

**Published:** 2025-11-12

**Authors:** Samuel E. I. Jones, Benjamin G. Freeman, Joseph A. Tobias, Steve J. Portugal

**Affiliations:** ^1^ Department of Biological Sciences, Georgia Institute of Technology, Atlanta, GA 30332, USA; ^2^ School of Biological Sciences, Royal Holloway University of London, Egham TW20 0EX, UK; ^3^ Institute of Zoology, Zoological Society of London, London NW1 4RY, UK; ^4^ Department of Life Sciences, Imperial College London, Ascot, Berkshire SL5 7PY, UK; ^5^ Department of Biological Sciences, University of Oxford, Oxford OX1 3RB, UK

**Keywords:** range determinants, tropical mountains, interspecific competition, physiological niche

## Abstract

Restricted elevational ranges are common across tropical montane species, but the mechanisms generating and maintaining these patterns remain poorly resolved. A long-standing hypothesis is that specialized thermal physiology explains these distributions. However, biotic factors such as habitat and interspecific competition have also been proposed to limit tropical species’ elevational ranges. We combined point-level abundances, respirometry-based measurements of metabolic rate, habitat surveys and playback experiments to simultaneously test these three hypotheses for four species of Central American cloud forest songbirds. Contrary to the physiological hypothesis, we found no evidence that thermoregulatory costs constrain species distributions. Instead, thermal conditions across each species’ elevational range remained well within sustainable limits, staying ≤65% of hypothesized thresholds for tropical birds, even at the highest elevations. By contrast, we found some support for a combined role of habitat and competition in shaping elevational ranges. In one related species pair, the dominant lower-elevation species appears restricted by microhabitat, while the higher-elevation species is likely prevented from expanding downslope by the presence of this congener. Taken together, we conclude that thermoregulatory costs are an inadequate explanation for elevational range limits of tropical birds at our site and suggest that biotic factors can be key in shaping these distributions. We provide a Spanish translation of the Abstract in the supplementary materials.

## 1. Introduction

What drives species’ distributions is a fundamental question in ecology that has direct application to forecasting species’ responses to ongoing anthropogenic change [1]. Tropical mountains contain significant environmental variation over comparatively small spatial scales [2], facilitating tests of the abiotic and biotic drivers of species ranges and minimizing any influence of dispersal constraints on distributions (see [1]). The pervasive pattern is that tropical montane species inhabit narrow elevational ranges, and sequences of related species occupying largely discrete ranges are common (‘elevational replacements’) [3–6]. Such elevational range patterns have attracted the attention of generations of ecologists [7–13], but despite long-standing interest, the mechanisms generating and maintaining these species distributions remain unresolved.

Empirical explanations for species’ elevational range limits in tropical mountains have largely converged on three hypotheses. First, the ‘thermal physiology’ hypothesis posits that tropical species have evolved thermal tolerances matched to their specific elevational ranges, such that ranges are limited by physiological constraints [14–16]. Second, the ‘ecotone hypothesis’ proposes that transitions between habitat boundaries dictate elevational ranges because of species-specific ecological specialism [3,17]. Third, the ‘competition hypothesis’ asserts that competition between ecologically similar species truncates their elevational ranges [8,18].

Historically, emphasis has been particularly placed on the hypothesis that thermal physiological barriers place limits on elevational distributions [14]. Specifically, a species moving to elevations outside its range would incur high energetic costs due to thermoregulation that would have negative fitness consequences [10]. Despite contrasting empirical evidence [19–21], specialized thermal physiology remains a central feature of explanations for elevational range restriction in the tropics [6,14–16]. In contrast, while case studies have provided evidence for habitat specialization [17,22,23] and interspecific competition [24–27], these hypotheses are less often considered to be general explanations (but see [28]).

Here, we test predictions arising from these three competing hypotheses by combining mechanistic field data on thermal physiology, microhabitat attributes and interspecific competition to predict species’ abundances across elevation in four Central American tropical cloud forest songbirds. We note that these hypotheses are not mutually exclusive, and that biotic and abiotic factors may interact to set range limits (e.g. [29]). We conducted physiological experiments to determine the energetic costs of thermoregulation, behavioural experiments to measure interference and interspecific competition, and detailed habitat measurements to quantify microhabitat across different slopes and ridgelines. We tested the following predictions. First, if the thermal physiology hypothesis holds, we expect the occupied elevational range to contain thermal conditions within which energy expended on thermoregulation is sustainable and that thermal conditions outside of this tolerable range will occur at higher or lower elevations unoccupied by the species. Second, if the ecotone hypothesis holds, we expect that microhabitat attributes, which are also likely related to species habitat requirements, would predict species’ abundances along the elevational gradient. Third, if the interspecific competition hypothesis holds, we expect an inverse relationship between the abundances of related species that segregate with elevation—note that we assess this last prediction only for one pair of closely related focal species.

## 2. Material and methods

### (a) Study site and fieldwork

**Figure 1.**
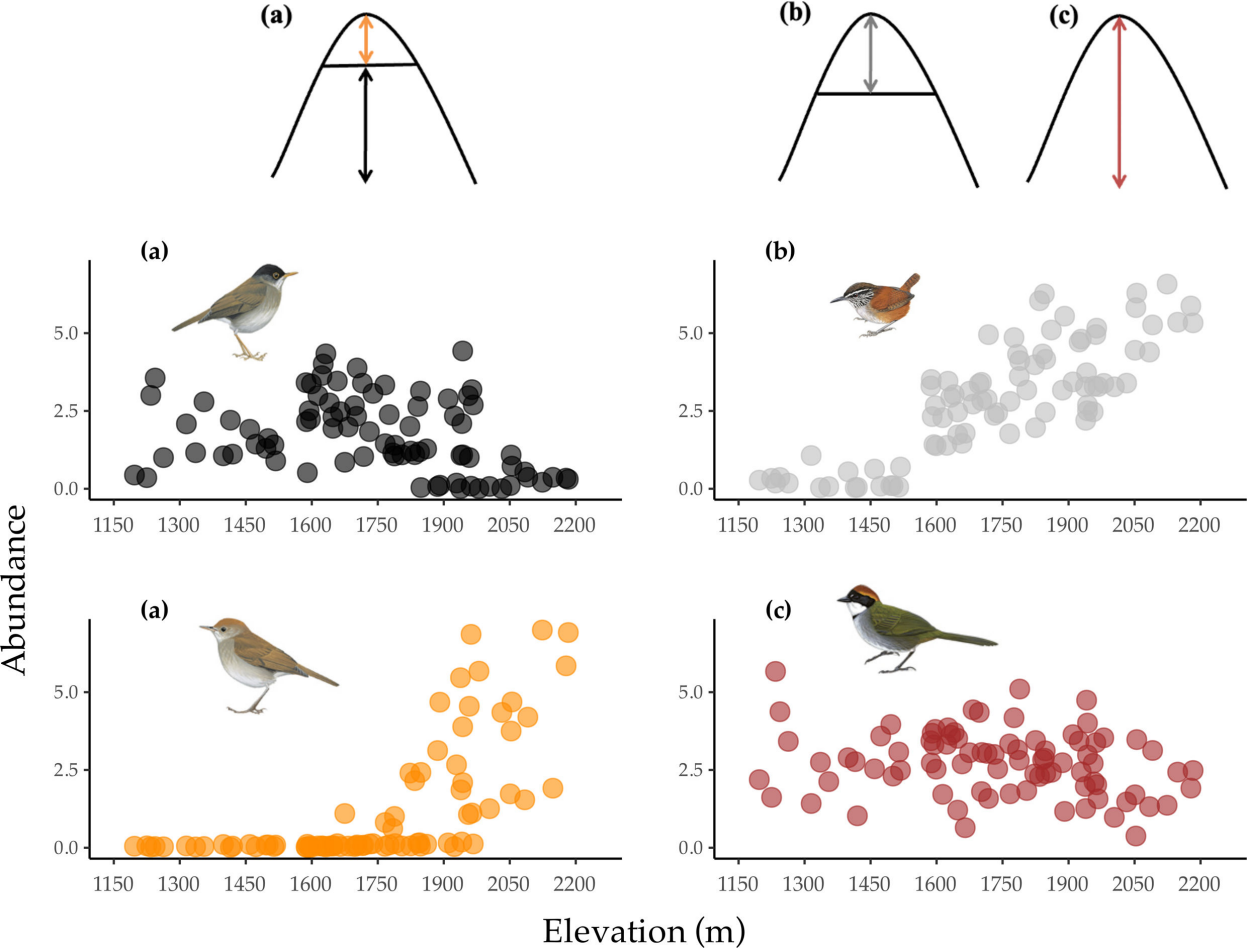
Elevational ranges and patterns of abundance of the four study species: (a) an example of elevational turnover where the lower-elevation black-headed nightingale-thrush *Catharus mexicanus* is ‘replaced’ by ruddy-capped nightingale-thrush *Catharus frantzii* at higher elevations, (b) grey-breasted wood-wren *Henicorhina leucophrys* is limited to mountaintops and (c) chestnut-capped brushfinch *Arremon brunneinucha* is distributed across the elevational range. The elevational range in this study was approximately 1200–2200 m.

We conducted fieldwork during the breeding season (June–August) in three consecutive years (2016−2018) in Cusuco National Park in the Sierra del Merendón, Honduras (approximately 15.552^o^ N −88.296^o^ W), the highest point of which is at 2242 m. We focused our work along a continuously forested gradient from 1197 to 2183 m, reducing any influence of land use on species’ abundances; lower elevations (below approximately 1100 m) are largely disturbed. Forests at our study site consist of broadleaf and mixed broadleaf and pine at elevations up to approximately 1900 m, with stunted elfin forest dominated by tree ferns at higher elevations [30]. In 2016, we surveyed the entire cloud forest bird community of Cusuco National Park and completed habitat surveys and playback experiments; we then collected physiological data in 2017−2018. We focused on four understory species (figure 1) that were common enough for us to obtain sufficient sample sizes for the behavioural and physiological aspects of our study. Survey and microhabitat data were collected along a number of transects that incorporated different ridgelines and slope aspects, allowing us to assess habitat and abundance across local variations in elevation.

### (b) Environmental data

**Figure 2.**
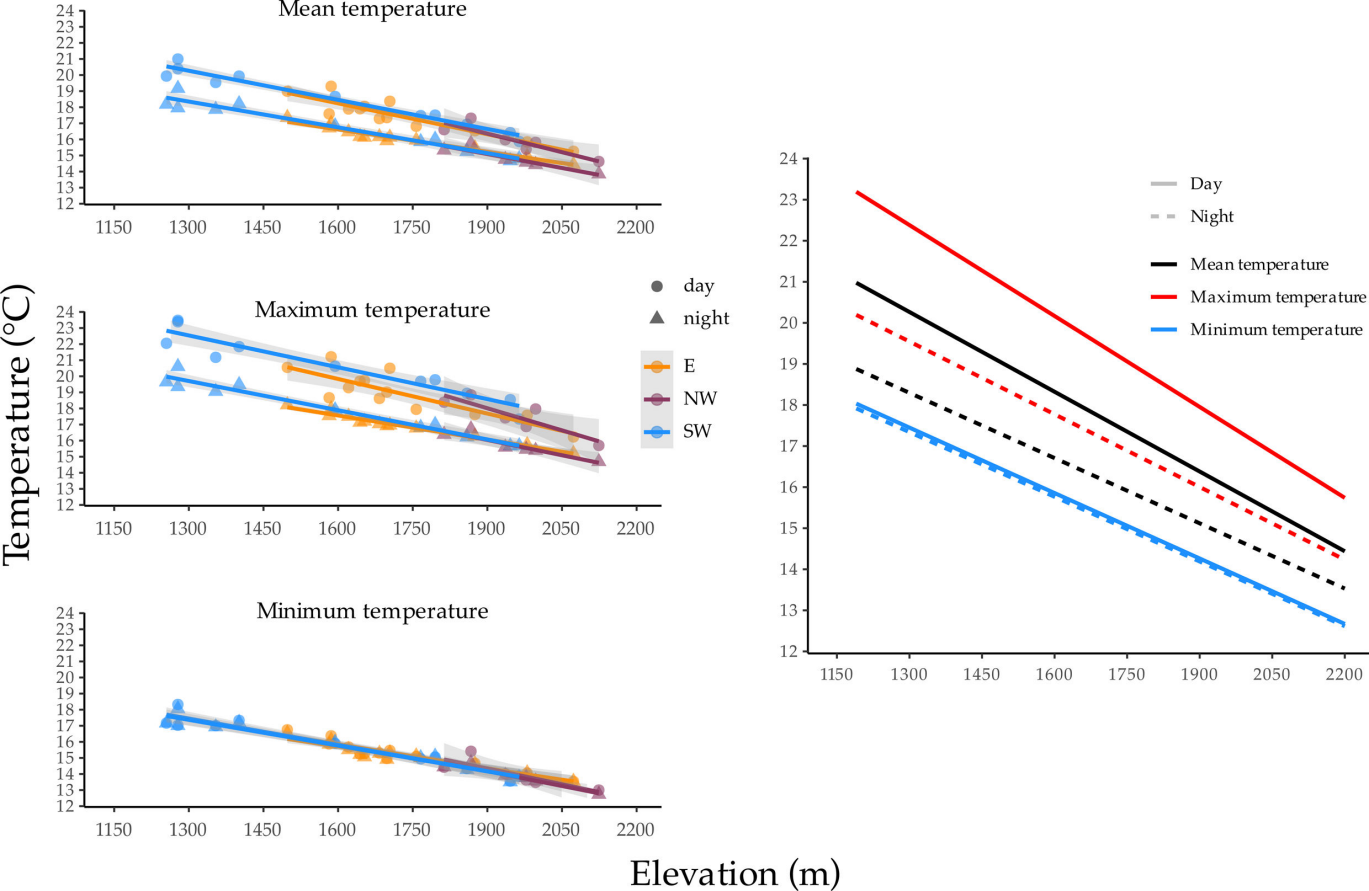
Environmental *T*_e_ variation and lapse rates across elevation, reflecting day and night-time means, mean maximum and mean minimum in centigrade (left panel), and modelled values across the total surveyed elevational gradient (right panel) used to predict thermal costs. Daily and nightly *T*_e_ lapse rates are comparable with increasing elevation, irrespective of slope aspect (see electronic supplementary material, table S1).

To characterize the thermal environment across elevation, we measured air temperature (environmental temperature: *T*_e_ (°C)) with remote loggers (*n* = 31) (HOBO Onset Pendant UA002-64 or EasyLog, EL-USB-2) across the elevational range surveyed, including each aspect of the mountain slope (figure 2). We attached loggers to the trunk of small trees or tree ferns approximately 1 m above the forest floor; these locations represent the microclimates that our focal species experience. Loggers recorded *T*_e_ at 30 min intervals for 24 h a day throughout the entire survey period; we then calculated mean *T*_e_, mean max *T*_e_, mean min *T*_e_ between sunrise (approximately 05.30) and sunset (approximately 18.30), and mean *T*_e_ between sunset and sunrise for each day. There was little evidence that slope aspect influenced temperature, so we did not include slope aspect in further analyses. As expected, *T*_e_ decreased linearly with elevation (all *r*^2^ between −0.84 and 0.96); we therefore regressed elevation on temperature and estimated *T*_e_ for each point count site on the basis of its elevation. For details of these analyses, see electronic supplementary material, tables S1 and S2.

### (c) Bird surveys

We completed point counts at 83 survey points that uniformly covered the elevational gradient. Each survey point was separated horizontally by at least 200 m to ensure sampling localities were independent [31]; points were situated along permanent study transects in strings of 3–8 points (see [30]). Following a 1 min settling period, we conducted 10 min point counts, recording the estimated distance to each detection (before data collection, we completed a distance calibration exercise between observers to improve precision). We surveyed points during peak vocal activity between 05.15 and 09.00, avoiding heavy wind or rain that might affect avian activity or detection. In total, we completed 351 point counts, visiting each survey point 1–7 times (mean 4.23 ± 1.98). To account for possible temporal biases in survey effort, we surveyed each transect at least once in reverse order (i.e. we visited the most distant points first). All survey data were subsequently truncated to a 70 m radius of survey points prior to analysis for the necessary requirements of abundance modelling (see §3).

### (d) Thermal physiology

We measured energy metabolism to establish energetic costs of focal species, then used these values in concert with environmental temperatures to model species’ thermoregulatory costs across elevation. We estimated metabolic rates across temperatures (to establish basal rates and metabolic rates below lower critical temperatures) using open-flow respirometry via a portable gas analysis system (FoxBox, Sable Systems, USA). Specifically, birds (*n* = 28 *Catharus mexicanus*; *n* = 11 *C. frantzii*; *n* = 8 *Henicorhina leucophrys*; *n* = 14 *Arremon brunneinucha*) were captured at dusk, fasted for at least 3 h and placed in a custom-built 20 cm^3^ Perspex chamber into which a constant airflow was supplied. We experimentally manipulated temperatures in this chamber by placing the chamber inside a modified cooler box fitted with a Peltier thermoelectric cooler module (AC-046; TE Technology, USA), capable of heating/cooling the interior of the cool box by a temperature controller (TC-48-20; TE 173 Technology, USA). Temperature plates were powered by an analogue 24V power bench (ALF2412; ELC, UK), externally powered by a portable generator, and exact temperatures inside the chamber were measured via a temperature logger (HOBO UA174 001-64; Onset, USA). We checked our respirometry set-up for leaks pre- and post-field seasons.

Ambient air pulled through chambers was dried (self-indicating silica gel; GeeJay chemicals, UK) and pulled through the chamber at 1000 ± 1 ml min^−1^; a sufficient flow rate at which O_2_ levels did not fall below 0.5% of natural levels, preventing hypoxia/hypercapnia. We used the same flow rates for all study species owing to their similar body sizes [20,32]. We measured and controlled air flow using a linearized mass flow meter internal to the FoxBox. Excurrent air from the chamber was dried again (silica gel) before entering the FoxBox, where O_2_ and CO_2_ content were recorded at 1 s intervals (Sable Systems ExpeData, Las Vegas, USA). We used a second air channel (outside ambient air, taken directly adjacent to the incurrent air to the chamber) as a reference, which was manually routed to the FoxBox following each temperature treatment (see below) to correct for drift in the analysers. Before sessions began, the O_2_ analyser was spanned to 20.95% (environmental O_2_ concentration) after gas measurements had stabilized.

We measured individual bird’s physiological responses to 1−4 temperature treatments per night, randomly choosing temperature treatments within five bands: 5–10°C, 11–16°C, 17–22°C, 23–28°C and 29–34°C (technological constraints prevented us from subjecting each bird to the same number of treatments per night). We used this randomized approach rather than a ramped profile to avoid potential temperature acclimation (e.g. [33]). We did not exceed 34°C during temperature manipulations as we were focusing on cold tolerance (the lower temperature limit of thermal neutrality), and 34°C was likely to be within thermal neutrality for our study species [20]. Once each temperature treatment was stable (±0.5°C of target temperature), we waited 45 min before beginning data collection of metabolic measurements. We then recorded data for approximately 20 min before changing to the next temperature treatment. Baselines of 7 min of ambient air were taken before each new temperature treatment was set (exact time intervals varied but were typically every 1−1.5 h). We took all metabolic data throughout the study during natural resting phases (night-time) and at least 4 h after capture to ensure birds were post-absorptive and only accepted metabolic data if birds were quiescent, consistent with similar studies (e.g. [34]). Respirometry sessions typically finished by 03.00; we released birds at the site of capture the following morning.

We measured how decreasing temperatures affected metabolic rates by estimating: (i) the inflection temperature (lower critical limit of thermoneutrality: *T*_lc_) at which each species began thermoregulation (i.e. an increase in metabolic rate), (ii) the slope of this relationship (estimated minimum thermal conductance: *C*_min_), and (iii) estimated basal metabolic rate above *T*_lc_ (BMR). This method can underestimate minimum *T*_lc_ [35] because we did not measure body temperatures, but this allowed an approximation of the energetic costs of thermoregulation below *T*_lc_. We focused only on cold tolerance to test the hypothesis that cold limits place restraints on upper limits of species distribution as they move upslope into cooler elevations [20]. We previously established thermal profiles for each species and reused these data here; for more details on methods and the analytical processes in establishing thermal models, see [32].

### (e) Estimating the fundamental physiological niche

We predicted thermoregulatory costs associated with living at different elevations for each focal species by using modelled values of estimated minimum thermal conductance (*C*_min_), lower critical temperature (*T*_lc_) and BMR. For example, if *T*_lc_ was 18°C, then temperatures greater than this would incur no energetic cost of thermoregulation, but temperatures below it would incur costs dependent on the slope of *C*_min_. Using these thermoregulation models (electronic supplementary material, figure S1), we predicted the metabolic rate for each species in multiples of BMR (see below) at the location of each temperature logger for the full time between sunset and sunrise per calendar day. We then averaged this value for the entire field season at the elevation of each temperature logger and predicted metabolic rate across the complete elevational range by fitting a linear model in the same manner as *T*_e_ (see §2b).

We mapped the estimated fundamental physiological niche to elevational ranges by considering thermoregulatory costs across elevation, assuming that birds cannot persist at elevations where they consistently breach the upper limits of their sustainable energy use (where energy was expended on the costs of thermoregulation at temperatures below *T*_lc_). We conservatively defined the upper limit of sustainable energy use expended on thermoregulation as 2 × BMR [10]. This is lower than temperate species, where sustained periods of energetic expenditure in excess of 4 × BMR are suggested to be above sustainable limits [36], but BMR is lower in tropical species than in temperate species [34,37], which may reflect a lower energetic ‘ceiling’ for tropical birds [10]. We predicted thermoregulatory costs only for night hours for two reasons: (i) our physiological data were only available from birds measured at night-time, and thus are directly comparable to field data, (ii) because body temperatures were not measured in our physiological study, it was not possible to accurately predict daytime costs using standard conversions of daytime body temperatures (e.g. [20]). Because night-time *T*_e_ invariably represents the lowest *T*_e_, our method models the most energetically demanding conditions for thermoregulation. We did not detect any thermoregulatory costs for *C. frantzii* across all temperatures tested, meaning that this species has effectively no costs to thermoregulation across the temperature range we studied. We therefore assigned a value of ‘1’ (i.e. BMR) for all modelled temperatures across elevation for *C. frantzii*.

### (f) Habitat surveys

We quantified habitat attributes by measuring vegetation within a 20 m^2^ plot surrounding each survey point. We quantified the following habitat attributes: percentage density of broadleaf, tree ferns or palm trees, understorey density, and leaf-litter depth/soil density. We quantified percentage densities of each tree type as the proportion of the cumulative diameter of all trees in each plot. This approach accounts for size differences between trees to better reflect microhabitat (e.g. broadleaf trees had larger diameters than tree ferns or palms). We measured mean understorey density within 3 m of the forest floor using the vertical ‘touch pole’ technique [38]. Here, we recorded the number of vegetation ‘touches’ on each 50 cm section of a 3 m pole stood at 1 m intervals along a bisecting line splitting the 20 m^2^ plot into quarters (max score at a given point = 6). The mean score across the 40 points (20 on each bisecting line) comprises an index of understorey density, with higher scores representing a thicker understorey and lower scores a more open understorey. Finally, we calculated a ‘forest floor’ index by soil depth and leaf-litter depth in five 2 m^2^ quadrats situated in the centre, and in each quarter of the bisected plot. Leaf-litter depth was measured by pushing a steel ruler through the leaf-litter until it hit topsoil; then measured as the height of the highest dead leaf in each quadrat. We measured soil density by dropping a standardized 1 kg spherical cone weight (cone end facing downwards) from 1.5 m height onto a patch of earth cleared of leaf litter and measured from the topsoil to the centre (deepest part) of the impact depression (units = mm). Leaf-litter depth and soil density variables were positively correlated, so we used values from the first axis of a principal component analysis ‘forest floor’ index; higher scores represent thicker/deeper forest floors. We selected variables that most obviously change across elevation, representing transitions between forest types at our field site [30,39], that our study species occupy, and that are known to be influential for tropical forest insectivores [40]. Our approach differs slightly from the classic ecotone hypothesis (where species range boundaries are predicted to coincide with elevation associated with major habitat transitions), but because such transitions are typically more gradual in tropical mountains and can differ across different ridges and slopes, our approach is effectively the same.

### (g) Interspecific competition

We previously established that the interaction between *C. mexicanus *and *C. frantzii* is asymmetric, with *C. mexicanus* showing interspecific territoriality towards its higher-elevation congener at elevational range limits [39]. Briefly, we measured interspecific competition between these two species by mapping territories and then performing reciprocal behavioural playback experiments on *C. mexicanus* (*n* = 29) and *C. frantzii* (*n* = 18) around their shared elevational range border. We did not perform playback experiments on our other two focal species because they lack abutting range limits with related elevational replacement species at our study site that might be putative competitors. We conducted playback experiments with three treatments (conspecific, heterospecific and control); each treatment consisted of 8 min trials (3 min of playback followed by 5 min of observation), broadcasting playback from a speaker placed in the centre of a given territory. We measured five behavioural response variables to each treatment, measuring only behaviours that occurred within 15 m of the speaker (closest approach to the speaker, latency to approach, latency to vocalize, number of vocalizations in response to playback and time spent within a 15 m radius of the speaker). This 15 m radius represents the greatest distance it was feasible to observe the focal bird in the forest understory. For further details on these experiments, see [39].

Informed by these data, we calculated a ‘distance to congener’ for each point count location by measuring the distance in metres to the closest territory of both *Catharus* species. This point-level measure incorporates intricacies of *Catharus* distribution at our study site, where the two *Catharus* species are always elevationally parapatric at the local scale, but there are ridge-to-ridge variations in the elevation of their contact zone such that at the entire study site scale they have slightly overlapping elevational distributions (e.g. figure 1). Using this approach allows us to incorporate the exact interspecific contact zone (see [38]), rather than assigning distance to interspecific contact on elevation alone.

## 3. Statistical analysis

### (a) Modelling bird abundances across elevation

We modelled site-level abundances for each species in ‘R’ [41] using *N*-mixture models in the *unmarked* package [42]. *N*-mixture models are a class of hierarchical model that correct for detection imperfections using repeated count data [43]. Our data include both true and false absences of detection, where a species was genuinely not present (e.g. above its elevational range limits), or was present but not detected (e.g. a non-singing bird). We thus used zero-inflated Poisson *N*-mixture models as a default to model both detection disparities, although we also fitted Gaussian models and assessed fits, respectively. Because *N*-mixture models are hierarchical, they simultaneously model Poisson-distributed abundance of a species at a survey site corrected for zero inflation and the probability of detecting the species given its true abundance. For the detectability aspect of the model, we included minutes since midnight (of the survey visit), ordinal day (days since 1 January) and observer (of four observers who undertook surveys) for each species model. These two variables represent possible differences in detection probability associated with song timing at dawn or variation in singing intensity in the breeding season.

We fitted models in a two-step process (e.g. [44]), where we first determined the importance of detection covariates for each species before modelling the abundance covariates. To do this, we fitted the detection probability covariates against an intercept-only model for abundance covariates, and then used the highest-ranking model with top detection covariate(s) from this phase for posterior modelling of abundance (electronic supplementary material, table S3). To determine the best detection covariates, we ranked models based on the Akaike information criteria corrected for small samples (AICc) using the *AICc modavg* package [45].

### (b) Testing hypothesized factors limiting elevational ranges

After establishing the best-fitting detection covariates per species, we included abundance covariates to test our hypotheses. For each species, we fitted each habitat variable, as well as thermoregulatory costs. We did not include thermoregulatory cost for *C. frantzii* because there was no evidence for thermoregulatory costs for this species. To test the interspecific competition hypothesis for *C. frantzii*, we included distance to *C. mexicanus* as a quadratic polynomial term. We did not include this competition term for the *C. mexicanus* model in our main results because we previously documented that *C. mexicanus* is behaviourally dominant [39]; however, we do include this model iteration in the electronic supplementary materials for completeness to explore possible evidence for other forms of competition (e.g. exploitative competition). We scaled all abundance variables so that magnitudes of change were comparable, log_10_-transformed understorey density so that it was normally distributed, and checked saturated models for each species for over-dispersion and goodness of fit using chi-square (χ^2^) and sums of squares statistics from 10 000 bootstrap samples. Our model for *C. frantzii* was slightly overdispersed, which is unlikely to strongly influence parameter estimates but may overestimate precision (see [43]).

We evaluated predictions of our hypotheses by examining model parameter estimates. For *C. frantzii*, we also assessed the strength of interspecific competition by assessing two model iterations by comparing AICc weights of a full model with all abundance covariates to a reduced model that lacked the distance to closest *C. mexicanus* term.

## 4. Results

### (a) Elevational ranges

We documented 310 records of *C. mexicanus*, 63 of *C. frantzii*, 379 of *H. leucophrys* and 203 of *A. brunneinucha*. We recorded *C. mexicanus* between 1234 and 2053 m (although all but five records were below 1960 m); *C. frantzii* between 1823 and 2183 m; *H. leucophrys* between 1315 and 2183 m (all but two records were above 1587 m); and *A. brunneinucha* across the entire elevational range surveyed. For point-level abundance estimates, see figure 1.

#### (i) Physiology

**Figure 3.**
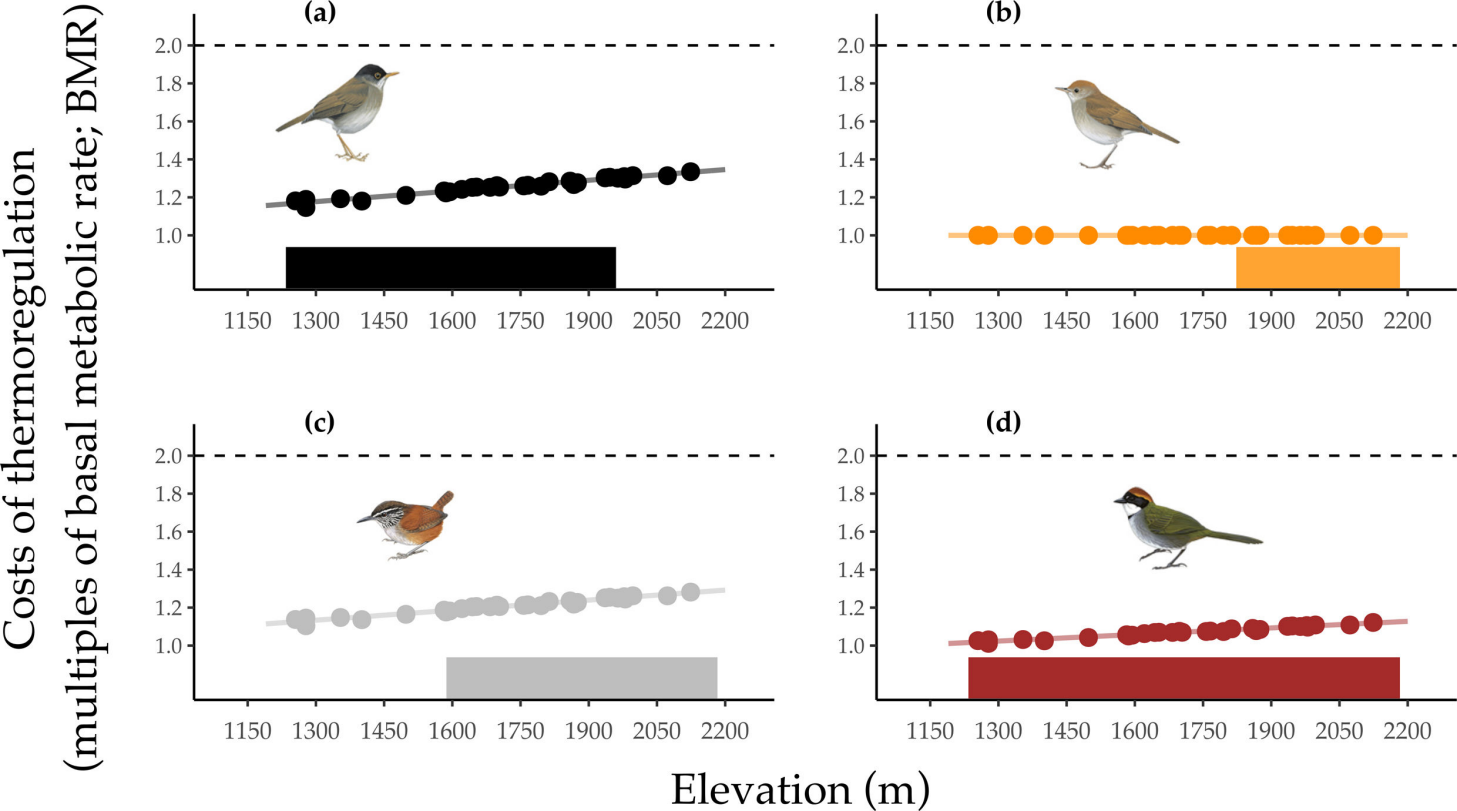
Predicted energetic costs of metabolic heat production (in multiples of basal metabolic rate (BMR)) against elevation and elevational range occupation in the four study species: (a) black-headed nightingale-thrushes *Catharus mexicanus,* (b) ruddy-capped nightingale-thrushes *Catharus frantzii*, (c) grey-breasted wood wrens *Henicorhina leucophrys* and (d) chestnut-capped brushfinches *Arremon brunneinucha*. Solid bars display the typical elevational range of each species. Points represent predicted mean nightly thermoregulatory costs at each temperature logger; we used these points to make continuous predictions across all point count locations, shown with a trendline. The dashed line at 2 × BMR displays the threshold above which the energetic costs of metabolic heat production are expected to be unsustainable [10].

We found no evidence that *T*_e_ at high elevations in our study site represented conditions at which thermoregulatory costs (>2 × BMR) were unsustainable for any of our focal species (figure 3). Even at the highest elevations surveyed, mean thermoregulatory costs (in multiples of BMR) were minor (1–1.3) for all species (*C. mexicanus* = 1.34, *H. leucophrys* = 1.29*, A. brunneinucha* = 1.13*, C. frantzii* = 1). For example, the mean thermoregulatory cost for *C. mexicanus’* high elevation limit (1960 m.a.s.l.) was 1.3 × BMR. Indeed, when we projected species’ thermoregulatory costs onto elevations above our study site (2242 m), we found that they would not breach mean nightly thermoregulatory costs of 2 × BMR until elevations thousands of metres above the species’ observed high elevation limits (all >5600 m)—a point at which metabolic power output would be affected by other abiotic features (e.g. air density) than environmental temperature. We did find a positive relationship between the abundance of *H. leucophrys* and metabolic cost, although this is a product of gradual increases in abundance of that species at higher elevations, covarying with declining temperatures (and thus increased metabolic costs) (electronic supplementary material, table S4).

### (b) Habitat

**Figure 4.**
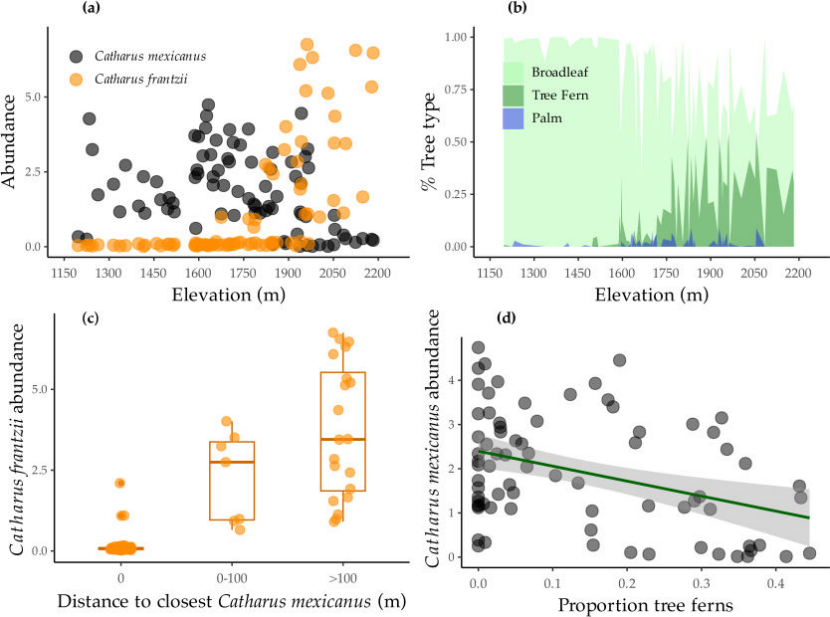
Relationships between *Catharus* nightingale-thrushes and elevation, and biotic features influencing elevational distributions: (a) abundance relationships across elevation in both species, (b) proportions of dominant vegetation types across elevation, (c) abundance of ruddy-capped nightingale-thrush *Catharus frantzii* in proximity to contact with black-headed nightingale-thrushes *Catharus mexicanus* and (d) black-headed nightingale-thrush *Catharus mexicanus* abundance in relation to tree ferns, typical of higher elevations (with regression to visualise the significant relationship). Collectively, this is consistent with competitive exclusion, where ruddy-capped nightingale-thrush *Catharus frantzii* are restricted to higher elevations by competition from the dominant black-headed nightingale-thrush *Catharus mexicanus,* which are restricted to lower elevations by habitat selection.

We found mixed evidence for the role of habitat in predicting elevational abundance patterns. *Catharus mexicanus* was negatively related to tree fern-dominated forests found at higher elevations (estimate = −0.476*, p* = 0.022), but positively related to proportions of palm (estimate = 0.365, *p* ≤ 0.001) (figure 4; electronic supplementary material, table S4). For its higher-elevation congener (*C. frantzii*), however, we found little influence of habitat preferences on abundance (all habitat variables *p* ≥ 0.24; electronic supplementary material, table S4).

For the remaining two species, we found no evidence that specific habitat types influenced patterns of elevational abundance of *H. leucophrys* (all habitat variables *p* ≥ 0.44; electronic supplementary material, table S4) or *A. brunneinucha* (all habitat variables *p* ≥ 0.25; electronic supplementary material, table S4).

### (c) Competition

We tested the competition hypothesis for one species pair: *C. mexicanus/frantzii*. Here, we found evidence in support of interspecific competition limiting elevational abundance patterns of *C. frantzii*; abundance increased nonlinearly with increasing distance from *C. mexicanus* (estimate = 9.800, *p* < 0.001) (figure 4; electronic supplementary material, table S4). Similarly, the model with the competition term performed better (AICc = 266) than one without it (AICc = 275.67). Taken together, this suggests that competitive exclusion is the most important factor driving the elevational abundance of *C. frantzii*, in contrast to *C. mexicanus* (see above).

## 5. Discussion

We found no evidence to suggest that physiological constraints limit the elevational distributions of the tropical montane birds we studied. In contrast, we found some positive evidence that biotic factors—habitat specialization and interspecific competition—can shape species’ abundances and elevational ranges. Specifically, we found that a combination of habitat and competition appears to limit the ranges of a pair of elevational replacement *Catharus* thrushes: we interpret our data to indicate that competition from the lower-elevation species restricts the upper elevation species’ range, while habitat specialization prevents the behaviourally dominant lower-elevation species from expanding to higher elevations.

### (a) Physiological limits to elevational ranges

Our main physiological result is that each of our focal species is physiologically capable of living across the entire elevational gradient at our study site. In fact, our focal species would only experience challenging thermal conditions at elevations >5600 m (dependent on species), well above species’ observed high elevation limits (<3500 m in all cases) and even higher than the highest mountain in Central America (Volcán Tajumulco in Guatemala, 4220 m). For example, *C. frantzii* showed no thermal cost to living in cooler temperatures despite being restricted to the mountain top, and all other species could occur well above their predicted thermal range globally; the highest elevations that *A. brunneinucha* and *H. leucophrys* occur at are approximately 3500 m in the Andes [46,47], yet our predicted thermal ceilings of these species are 9753 and 6215 m, respectively. Though studies of the thermal physiology of tropical birds remain scarce, our results are consistent with previous studies [19,20,48]. Our predicted thermoregulatory costs (in multiples of BMR) were slightly lower in our study than in others [20], likely a product of only including higher-elevation species in our study, less precision of thermal conductance values in the absence of body temperature measurements and seasonally elevated rates of BMR in some species [32]. Nonetheless, collectively, these data strongly suggest that critical thermoregulatory limits do not limit the elevational ranges of tropical birds, as suggested by Janzen’s [14] predictions.

We recognize, though, that the full energetic costs of avian thermoregulation are a function of complex relationships between the environment, species physiology (e.g. body temperature) and other aspects of behaviour and condition (e.g. microclimate use and plumage) [49–55]. Our methodology does not provide this complete picture, which would require additional measurements of cold performance such as summit metabolic rate and measures of body temperature. Further, we emphasize that we measured thermal tolerances on adult birds only; it remains possible that the thermal specialization hypothesis applies to eggs or chicks, which are ectothermic throughout development, via physiological specialization at these early life stages or by specific nest structures that could confer thermal ‘refugia’ for sensitive nestlings [56]. We focused on species living only above 1000 m; species of tropical lowlands may face greater thermal challenges than those of higher elevations [20]. Nevertheless, available evidence increasingly suggests that the mechanistic relevance of physiological specialization to elevational ranges, at least for adult birds, is limited (see [15]).

There are four reasons why we believe our conclusion that thermal physiology does not limit our study species’ elevational distributions to be robust. First, *T*_e_ in forests are typically ‘stable’ (i.e. little wind or influence of insolation [10]) and thus unlikely to affect thermal budgets for understorey forest species. Second, because night-time (cooler) hours represent the greatest thermal challenges, it is unlikely our results would change if we also measured thermoregulatory costs during the daytime. Daytime thermal conductance and body temperatures are typically greater than at night [57–60], but still incur less energetic demands [20] because of reduced heat loss to the environment during the day. Third, we calculated energetic costs during the breeding season, which is likely to be the more physiologically stressful season for these resident birds. Our previous research for three study species (excluding *C. frantzii*) documented that metabolic rates remain constant or downregulate (for *C. mexicanus*) during the cooler non-breeding season [32], implying that each species lives within sustainable thermal limits throughout the year. Fourth, the 2 × BMR threshold is the most conservative estimate suggested by [10]; birds may be able to sustain energetic costs in the short or median term of up to 4−7 × BMR [61,62]. Our values all fell considerably below this figure, demonstrating that each species was well within its metabolic scope. Indeed, the greatest single predicted night-time thermoregulatory cost incurred by any species at the field site in the breeding season (*C. mexicanus*; 1.52 × BMR) was still substantially below suggested unsustainable limits.

### (b) Biotic limits to elevational ranges

We found evidence that biotic variables interact to restrict the elevational ranges for two of our species: specifically, the combination of competition and habitat appears to shape the mutual range limit of the *C. mexicanus*, *C. frantzii* species pair. Habitat mediates the elevational range of the lower-elevation *C. mexicanus* but not for the higher-elevation *C. frantzii*, while *C. mexicanus* is competitively dominant over *C. frantzii* (figure 4). We interpret these results to mean that *C. mexicanus* selectively avoids tree fern-dominated forest that is common at higher elevations (figure 4), while interference competition from *C. mexicanus* restricts *C. frantzii* to living only at higher elevations with habitat that *C. mexicanus* avoids.

We suggest that the interaction between competitive ability and habitat requirements may be a common phenomenon influencing species’ ranges along elevational gradients. Several studies have documented interspecific aggression between closely related species of tropical mountain birds and interpreted these findings as evidence that competition can limit ranges (e.g. [26,27]), but these behavioural interactions are typically asymmetric [63]. What then prevents a behaviourally dominant species from expanding its range into that of the subordinate species? Trade-offs are a long-standing theme in ecological research, including on species’ ranges (e.g. [29]). We speculate that our case example of *Catharus* thrushes illustrates a broader pattern in tropical montane species replacements, consistent with evidence of habitat segregation between congeneric species pairs in the tropical lowlands [64]. To test this hypothesis mechanistically would require experimental habitat manipulation or translocation; studies that combine measurements of interference, interspecific competition *and* habitat features (e.g. productivity) are an important first step to better explore potential mechanisms underlying these combined factors. Finally, while we make these suggestions based on prior knowledge of interspecific territoriality between the *Catharus* species from our study, it remains possible that subordinate species could exhibit exploitative competition pressure over dominant species subsequently influencing range limits ([65], electronic supplementary material, table S5).

We did not find evidence for habitat influencing range patterns for three species in our study. For the one species (*A. brunneinucha*) that occurs across our whole study gradient, this is perhaps unsurprising. However, this was less expected for *H. leucophrys*, which lives only >1600 m at our study site and lacks an abutting range boundary with a congener (a ‘no man’s land’ of over 300 m of elevation separates *H. leucophyrs* from the lower-elevation *H. leucosticta* at our study site). This may reflect a lack of precision in our habitat variables, where fine-scale features (e.g. aerial leaf-litter) are important [66]. Alternatively, other features we did not measure may drive range limits such as nest predators [67] or parasites [10,68].

Understanding how abiotic and biotic factors limit species’ elevational ranges is important to forecasting potential elevational upslope range changes in the face of climate change [69]. For example, we speculate that upslope range shifts in *C. mexicanus* will not be limited directly by temperature, but instead may be modulated by habitat boundaries. If so, this would be an example of how upslope shifts in birds may be limited by plants, possibly explaining why observed upslope shifts often lag behind rates of warming [70,71], as slow changes of habitat transition may slow the process of range shifts [72].

## 6. Conclusions

Taken together, we conclude that biotic factors are more likely to limit the elevational ranges of species in our study than physiological determinants. Given the consistency of our physiological results to previous studies from multiple continents, it seems unlikely that thermoregulatory barriers generally place limits on elevational ranges in birds [20,66,67]. However, exactly how or what biotic factors influence different species may be site and/or species dependent. Accordingly, we echo the comments of previous authors (e.g. [10,20]) in emphasizing the importance of site-/species-specific studies to better interrogate the importance of elevational range determinants across complex montane systems. Whether this pattern can be applied beyond birds remains unanswered; the only study we are aware of that has tested multiple mechanistic hypotheses in ectotherms found stronger support for biotic over physiological factors for lizards in the Bolivian Andes [21].

The importance of understanding the mechanisms that drive and maintain elevational ranges cannot be overstated. Upslope movements and local extirpations in montane species have been documented throughout the tropics, including at our study site [73–77], in line with projections that tropical montane climates may be severely altered by environmental change within a century [78]. Without mechanistic assessments of species range occupation, we lack the fundamental data from which to make informed predictions of how species will respond to environmental change in the world’s most biodiverse regions.

## Data Availability

All data and scripts for this paper available at [79]. Supplementary material is available online [80].
